# Protanopia (red color-blindness) in medaka: a simple system for producing color-blind fish and testing their spectral sensitivity

**DOI:** 10.1186/s12863-017-0477-7

**Published:** 2017-02-06

**Authors:** Noriko Homma, Yumi Harada, Tamaki Uchikawa, Yasuhiro Kamei, Shoji Fukamachi

**Affiliations:** 1 Department of Chemical and Biological Sciences, Japan Women’s University, Tokyo, Japan; 2 National Institute for Basic Biology, Aichi, Japan; 3 School of Life Science, The Graduate University for Advanced Studies (SOKENDAI), Aichi, Japan; 4 Laboratory of Evolutionary Genetics, Department of Chemical and Biological Sciences, Japan Women’s University, Mejirodai 2-8-1, Bunkyo-ku, Tokyo, 112-8681 Japan

**Keywords:** Color-blind medaka (*Oryzias latipes*), Spectral sensitivity, Cone opsin, Long-wavelength sensitive (LWS)

## Abstract

**Background:**

Color perception is important for fish to survive and reproduce in nature. Visual pigments in the retinal photoreceptor cells are responsible for receiving light stimuli, but the function of the pigments in vivo has not been directly investigated in many animals due to the lack of color-blind lines and appropriate color-perception tests.

**Methods:**

In this study, we established a system for producing color-blind fish and testing their spectral sensitivity. First, we disrupted long-wavelength-sensitive (LWS) opsins of medaka (*Oryzias latipes*) using the CRISPR/Cas9 system to make red-color-blind lines. Single guide RNAs were designed using the consensus sequences between the paralogous *LWSa* and *LWSb* genes to simultaneously introduce double-frameshift mutations. Next, we developed a non-invasive and no-prior-learning test for spectral sensitivity by applying an optomotor response (OMR) test under an Okazaki Large Spectrograph (OLS), termed the O-O test. We constructed an electrical-rotary cylinder with black/white stripes, into which a glass aquarium containing one or more fish was placed under various monochromatic light conditions. The medaka were irradiated by the OLS every 10 nm, from wavelengths of 700 nm to 900 nm, and OMR was evaluated under each condition.

**Results:**

We confirmed that the *lws* ^*−*^ medaka were indeed insensitive to red light (protanopia). While the control fish responded to wavelengths of up to 830 nm (λ = 830 nm), the *lws* ^*−*^ mutants responded up to λ = 740 nm; however, this difference was not observed after adaptation to dark: both the control and *lws* ^−^ fish could respond up to λ = 820 ~ 830 nm.

**Conclusions:**

These results suggest that the *lws* ^−^ mutants lost photopic red-cone vision, but retained scotopic rod vision. Considering that the peak absorption spectra (λ_max_) of medaka LWSs are about 560 nm, but the light-adapted control medaka could respond behaviorally to light at λ = 830 nm, red-cone vision could cover an unexpectedly wide range of wavelengths, and behavioral tests could be an effective way to measure spectral sensitivity. Using the CRISPR/Cas9 and O-O systems, the establishment of various other color-blind lines and assessment of their spectra sensitivity could be expected to proceed in the future.

**Electronic supplementary material:**

The online version of this article (doi:10.1186/s12863-017-0477-7) contains supplementary material, which is available to authorized users.

## Background

Color perception is crucial for animals to survive, for example, in food choice, avoidance of predators (mimicry), and successful selection of a reproductive partner. Colors are virtual images provoked in the brain according to a relative balance of light at multiple wavelengths received in the retina. In retinal photoreceptor cells, most vertebrates other than mammals have one type of opsin in rods for brightness vision (rhodopsin 1; RH1), and four spectral classes of opsins in cones for color vision (short-wavelength sensitive 1, SWS1; short-wavelength sensitive 2, SWS2; rhodopsin 2, RH2; and long-wavelength sensitive, LWS; which are sensitive to violet, blue, green, and red lights, respectively) [1].

In the evolution of fish color vision, the multiplications of the *cone-opsin* genes had important roles for environmental adaptation in nature [2–4]. For example, duplication of the *SWS1* and *SWS2*, and quadruplication of *RH2* occurred in zebrafish [5, 6]. In medaka, such multiplications resulted in two *SWS2*s (*SWS2a* and *SWS2b*), three *RH2*s (*RH2a*, *RH2b*, and *RH2c*), and two *LWS*s (*LWS a* and *LWSb*) [7]. These paralogous cone opsins often have distinctive absorption spectra via sub-functionalization, and may be responsible for fine-tuning of their color perception, enabling fish to flourish in the colorful underwater world under various light conditions.

In past decades, spectral sensitivities of cone opsins of various fish species were measured using different methods. Some studies showed electrophysiologically measured S-potential in the retina or electroretinography (ERG) [8–10], while others showed biochemically measured absorption spectra of reconstituted opsin molecules in vitro [11, 12, 13]. In medaka, peak absorption spectra (λ_max_) of reconstituted cone opsins with 11-cis retinal [7] were 356 nm (SWS1), 439/405 nm (SWS2a/b), 452/516/492 nm (RH2a/b/c), and 561/562 nm (LWSa/b). However, the actual spectral sensitivity of each pigment in vivo has not been elucidated. That is, measured spectral sensitivity may not necessarily be identical between the molecular (or electrophysiological) and behavioral levels.

To analyze behavioral spectral sensitivity, it is necessary to make a series of *cone-opsin* mutants. The loss of function in the SWS1, SWS2, RH2, or LWS opsin will cause violet, blue, green, and red color-blindness or color-weakness, respectively. Until recently, however, it has been difficult to make such color-blind fish lines because *cone-opsin* genes are small (about 350 amino acids) and an effective method for simultaneously deleting all *cone-opsin* paralogs has been lacking.

In addition, a behavioral test for spectral sensitivity needs to be developed. There have been a few tests for measuring spectral sensitivity of fish depending on phototaxis [14], feeding [15], or learning [16], but these tests require a large quantity of specimens, time to induce starvation, and prior training. Optomotor response (OMR) is another choice for testing the spectral sensitivity of fish in vivo [12, 17]. OMR is an instinctive response to follow moving images on the retina and is often applied to fish using rotating vertical black/white stripes. In 1998, Hasegawa utilized OMR to measure the spectral sensitivity of fish [12]. His experiment, which was conducted under monochromatic lights at λ = 400, 500, and 600 nm using the Okazaki Large Spectrograph (OLS; see Methods) and reported in Japanese, was worthy of note and had the potential to be further improved; however, it was obscured for nearly 20 years.

In this study, we established a systematic procedure for making color-blind medaka (*Oryzias latipes*) and testing their behavioral spectral sensitivity. The first color-blind line to be reported here is red color-blind medaka, the *lws* ^*−*^ mutant, in which all the paralogous *LWS* genes (*LWSa* and *LWSb*) were simultaneously mutated using the Clustered Regularly Interspaced Short Palindromic Repeat (CRISPR)/CRISPR-associated proteins 9 (Cas9) system [18–20]. We designed single guide RNA (sgRNA) from the consensus sequence of the paralogs and successfully introduced double-frameshift mutations. To confirm red color-blindness in the *lws* ^*−*^ mutants, we improved the behavioral assay conducted by Hasegawa [12]. Our OMR test under the OLS, named the O-O test, is simple, fast, and non-invasive without any training or immobilization, but can induce much faster responses and be conducted at any wavelength. Using this system, a series of various color-blind fish lacking one or more cone opsins could be generated, and their spectral sensitivity could be analyzed efficiently in vivo.

## Methods

### Fish lines and care

We used two medaka lines as a host (control) into which frameshift mutations were introduced by CRISPR/Cas9; the *color interfere* (*ci*) line with a mutation on the *somatolactin-alpha* (*SLα*) gene [21] and the transgenic *ci* line with a transgene that ectopically expresses SLα (Actb-SLα:GFP [22]). The reason we used *ci* and Actb-SLα:GFP (instead of wild type) for establishing the *lws* ^*−*^ line was because these medaka exhibited unique and conspicuous sexual preferences towards mates of the same strain [23]. Therefore, they could be an appropriate model for future studies of color perception in this species, as we have already confirmed that these preferences are based on skin color [24]. All fish were born and bred in laboratory aquariums. Light was provided by using ordinary fluorescent lamps for 14 h a day and water was circulated and filtrated at 27 °C. The fish were fed with hatched *Artemia* and flake food (TetraMin) five times a day.

### Preparation and microinjection of the *Cas9* mRNA and sgRNA

**Fig. 1:**
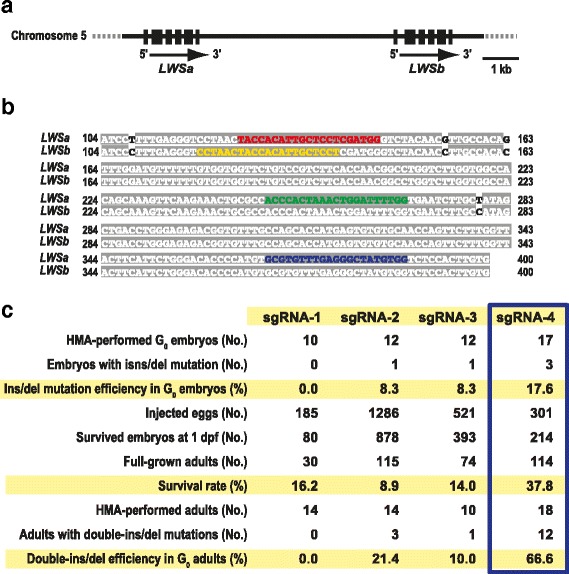
Detailed schemes for introducing double-ins/del mutations into the *LWSa* and *LWSb* loci and a summary of the results. (**a**) Genomic structures of the *LWSa* and *LWSb* loci. Coding exons are shown as solid boxes. (**b**) Alignment of the second exons of the paralogous *LWS* sequences. The A residue in the first exon, at which translation is initiated, is numbered as the first. Shaded white text indicates identical nucleotides. Only four nucleotides were different between the *LWS* genes. Red, yellow, green, and blue text indicate the target sequences of the sgRNA-1, 2, 3, and 4, respectively. (**c**) Details and efficiencies of inducing the double-ins/del mutations on the *LWSa* and *LWSb* genes in the G_0_ generation. The sgRNA-4 achieved the highest induction efficiencies in both the embryonic and adult stages (boxed)

The sgRNA expression vector (pDR274; Addgene plasmid 42250) was linearized with BsaI, electrophoresed in a 2% agarose gel, and purified using a Wizard SV Gel and PCR Clean-up System (Promega). Appropriately designed oligonucleotides were purchased from Hokkaido System Science or Life Technologies. A pair of complementary oligonucleotides were annealed in a buffer (40 mM Tris–HCl [pH 8.0], 20 mM MgCl_2_, and 50 mM NaCl) by heating at 95 °C for 2 min and then cooling slowly to 25 °C in 1 h. The double-stranded oligonucleotides were ligated into the linearized pDR274 vector. We verified appropriate orientation of the oligonucleotides by colony PCR and direct sequencing. Each sgRNA were designed to target both the *LWSa* and *LWSb* genes. The target sequences are shown in Fig. 1b.

The oligonucleotide-inserted pDR274 vector and the *Cas9* expression vector (hCas9; gifted by Prof. Zhang of Massachusetts Institute of Technology) were linearized with DraI and NotI, respectively, and the sgRNAs and the capped *Cas9* mRNA were synthesized using an AmpliScribe T7-flash Transcription Kit (Epicentre) and an mMessage mMachine SP6 kit (Life Technology), respectively. The synthesized RNAs were purified by RNeasy mini Kit (Qiagen). A mixed solution of the sgRNA (25 ng/μl) and the *Cas9* mRNA (100 ng/μl) were microinjected into the one-cell stage embryos of the *ci* or Actb-SLα:GFP lines using fine glass needles. A day later, dead embryos were removed, and those remaining were used for analysis and breeding.

### Identification of ins/del and frameshift mutations

Ins/del mutations in the target sequences were identified by heteroduplex mobility assay (HMA) as described elsewhere [19], except that we used 12% polyacrylamide gels for electrophoreses. Genomic DNA was extracted from 2 ~ 3-day-old embryos or the caudal fins of adults, and 100 ~ 150-bp genomic fragments, containing the target sequence, were amplified by PCR using appropriate primers. The PCR conditions were as follows; initial denaturation at 94 °C for 1 min, followed by 30 cycles of 98 °C for 20 s, 60 °C for 1 min, and 72 °C for 1 min. The amplified products were heated at 98 °C for 1 min and cooled to room temperature before electrophoresis.

To obtain F_1_ fish, the injected (G_0_) adults having the ins/del mutation in their caudal fins were mated with non-injected control fish. If inheritance of the ins/del mutation from the G_0_ to F_1_ embryos was detected by HMA, we re-amplified the target sequence into longer fragments by genomic PCR (primer sequences were; LWSa-F: 5′-tggttggattctaagggtttaaacg, LWSb-F: 5′-acctttgatataagcagctggaggt, and LWSa/b-R: 5′-cagttaccaaaacaacaccaaccat) and directly sequenced the products. By manually subtracting the wild-type sequence from the mixed electropherogram, we determined the ins/del sequences. The heterozygous F_1_ males and females that had identical double-frameshift mutations on the *LWSa* and *LWSb* genes were intercrossed to obtain F_2_ fish that are homozygous for the mutations (the *lws* ^*−*^ medaka) at the probability of 1/4.

### Reverse transcription-polymerase chain reaction (RT-PCR)

We extracted total RNAs from the whole eyes of adult fish using Isogen II (Nippon Gene), and synthesized cDNA using ReverTra Ace (Toyobo) and polyT primers. Primers for PCR were F: 5′-ttctgtccgtcttcaccaacg and R: 5′-caaagacgggaggtgcacac for the *LWS* paralogs (we did not discriminate *LWSa* and *LWSb* paralogs because they have highly similar coding and untranslated sequences), and F: 5′-gattcccttgaaacgaaaagcc and R: 5′-cagggctgttgaaagtctcaaac for the *beta-actin* (*Actb*) gene. Temperature conditions were 96 °C for 1 min, followed by 32 cycles of 98 °C for 20 s, 60 °C for 1 min, 72 °C for 30 s, and 72 °C for 10 min. Products were electrophoresed on a 3% agarose gel and bands were visualized by ethidium bromide staining and UV illumination.

### The optomotor response test under the monochromatic lights of the Okazaki Large Spectrograph, the O-O test

We constructed an electrical apparatus that can rotate a cylinder with inner vertical black/white stripes (a 1-cm wide black stripe every 3 cm) at a regular speed in both clockwise and counter-clockwise directions (see Fig. 4b). Inside the rotary cylinder, we placed a glass aquarium, which contains 1 ~ 5 juvenile or adult fish, and rotated the cylinder at 6 rpm under various monochromatic light conditions. Water depth was about 3 cm.

The OLS irradiates horizontal light with various wavelengths (but not necessarily with identical brightness; see Table 1). These monochromatic lights were vertically reflected by a mirror (“mirror1” in Fig. 4c) to illuminate the apparatus from the top. The monochromatic lights are parallel light, but a white plastic paper under the aquarium scatters the light to illuminate the inner stripes of the cylinder. We set another mirror beneath the aquarium (“mirror2” in Fig. 4c) and the ORCA-R2 Digital CCD camera (Hamamatsu photonics) to video-record the behavior of the fish as silhouettes on the plastic paper.

**Table 1:** Intensities of the monochromatic lights irradiated from the OLS

Wavelength (nm)	Photon flux density (μmol/m^2^s)^a^	Photon flux density (μmol/m^2^s)^b^	Half bandwidth
700	65	70	±5 nm
710	78	-	-
720	63	-	-
730	67	-	-
740	59	-	-
750	53	63	±4 nm
760	73	-	-
770	44	-	-
780	36	-	-
790	41	-	-
800	39	51	±4 nm
810	38	-	-
820	85	-	-
830	90	-	-
840	48	-	-
850	24	34	±4 nm

^a^Values measured by a light quantum meter (QTM-101; Monotech)

^b^Values measured by a spectroradiometer (S-2440C; Soma Optics) and calculated as integration between ±50 nm of wavelengths

The O-O test was performed at every 10 nm at wavelengths of λ = 700 nm to λ = 900 nm. We rotated the cylinder in both clockwise and counter-clockwise directions, and the direction was changed every 30 s, typically three times. We judged the irradiated light as visible to medaka when fish followed the rotating stripes immediately after changing the direction of cylinder rotation (within 10 s). We used night vision goggles (Noctovision NVR2015, NEC Corporation, Japan) that do not irradiate infrared light for the tests under invisible or nearly invisible light for humans.

### F_2_ screening using the O-O test at λ = 760 nm

By intercrossing heterozygous *lws* ^*−*^ (*lws* ^+2a+5b^, *lws* ^*−*1a-19b^, or *lws* ^*−*2a-19b^) mutants, we obtained F_2_ fish from juveniles (>12 mm of body length) to mature adults. One-fourth of these should be homozygous *lws* ^*−*^ mutants, but are indistinguishable by their phenotypes (e.g., shape, size, color, etc.) under ordinary breeding conditions. We individually evaluated their OMR at λ = 760 nm by the O-O test; OMR-positive fish should be wild type or heterozygous mutants, whereas OMR-negative fish should be *lws* ^*−*^ mutants. Then, we verified their genotypes by PCR and direct sequencing to judge our evaluations.

### The O-O test after light or dark adaptations

For light adaptation, we put the fish under ordinary fluorescent lamps (about 30 μmol/m^2^ s) for over 5 min. Then, the fish were immediately used for the O-O test as described above. In order to prevent the potential of the fish adapting to invisible lights (dark environment), we started the test with visible monochromatic light with relatively short wavelengths. After the test, the fish were light-adapted again by fluorescent lamps and then used for the next test at wavelengths 10 nm longer than that in the previous test. We repeated these procedures lengthening the wavelength each time until the fish stopped following the stripes.

For dark adaptation, we put fish in a wooden black box for over 2 h, prior to the O-O test. After dark adaptation, we wore night vision goggles, turned off all lights in the OLS room, set the aquarium in the cylinder, irradiated the monochromatic light, and performed the O-O test. We started the test at λ = 900 nm. We changed the direction of cylinder rotation, two times per test, before judging if the fish followed the stripes in order to minimize potential light adaptation via the invisible light. After a test, we returned the fish to the black box, turned on the lights in the OLS room, moved the entire apparatus to a position where monochromatic light was available at a wavelength 10 nm shorter and performed the next test. We repeated these procedures until the fish started following the stripes.

## Results

### Simultaneous introduction of frameshift mutations into the medaka *LWS* paralogs, *LWSa* and *LWSb*

The medaka *LWSa* and *LWSb* genes are tightly linked on chromosome 5 (Fig. 1a) and the alignment of the paralogous sequences revealed that 98.8% (1,061/1,074) of the coding regions of *LWSa* and *LWSb* were identical (see Fig. 1b). We determined four target sequences for CRISPR/Cas9 in the second exon that encodes 35 ~ 134 amino acids of the LWSa/b protein corresponding to the first three transmembrane domains. We also confirmed that there were no similar sequences (potential off-target sequences) in the medaka genome by the Medaka Pattern Match Tool (http://viewer.shigen.info/medakavw/crisprtool/).

**Fig. 2:**
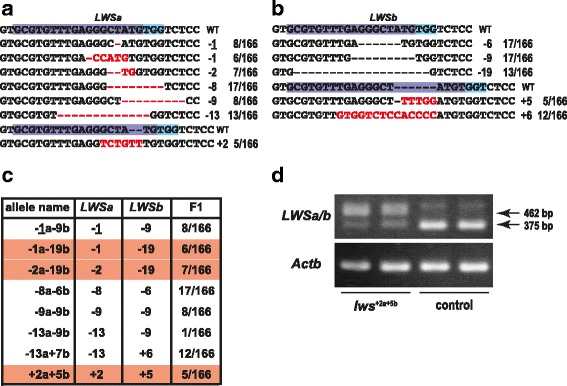
The ins/del mutations. (**a**) and (**b**) Summary of the ins/del mutations detected in the *LWSa* (**a**) and *LWSb* (**b**) alleles. The target sequence of sgRNA-4 is highlighted in purple and blue. The inserted or deleted nucleotides are indicated by red characters or red hyphens, respectively. The total number of ins/del nucleotides and the number of F_1_ fish that inherited each mutation (of the 166 F_1_ fish analyzed) are shown on the right. (**c**) Summary of the double-ins/del alleles inherited to F_1_ fish. The allele names are based on the total number of ins/del nucleotides in each gene; e.g., “+2a + 5b” indicates two and five nucleotides were inserted in the *LWSa* and *LWSb* genes, respectively. Thus, three of these eight double-ins/del mutations (highlighted in red) cause double frameshifts on both the *LWSa* and *LWSb* genes. (**d**) RT-PCR images using the eyes. Because of the high sequence similarity between the *LWSa* and *LWSb* genes (see Fig. 1b), we did not discriminate between them for RT-PCR analysis, and an identical pair of primers was used for simultaneous amplification of the *LWSa* and *LWSb* transcripts. The primers sandwich an intron (87 bp) and, therefore, bands at 375 bp and 462 bp are products from cDNA and genomic DNA, respectively. Considering that the 462-bp products are preferentially amplified in *lws* ^*+2a+5b*^ (while they are not amplified at all in control), transcription of *LWSa/b* in the *lws* ^*+2a+5b*^ mutants seems to be very weak in comparison with that in the control fish. A larger band (>500 bp) found in all lanes could be non-specific

We designed and synthesized four types of sgRNAs targeting Exon 2, and microinjected them individually to fertilized eggs. The induction efficiencies of double-ins/del mutations on the *LWSa* and *LWSb* genes by sgRNA-1 ~ 4 were 0.0%, 8.3%, 8.0%, and 17.6% in G_0_ embryos, and 0.0%, 8.9%, 10.0%, and 66.6% in G_0_ adults, respectively (Fig. 1c). Some of the G_0_ adults inherited the ins/del alleles to F_1_, and we found seven and five types in *LWSa* and *LWSb*, respectively (Fig. 2a and b) in eight combinations, three of which were double-frameshift mutations (Fig. 2c). Homozygotes for these double-frameshift mutations could be successfully isolated by intercrossing the F_1_ fish, and we designated these *lws* ^*−*^ mutants, *lws* ^*−*1a-19b^, *lws* ^*−*2a-19b^, and *lws* ^+2a+5b^, according to the number of ins/del nucleotides on the *LWS* paralogs. Reduction of *LWS* transcripts in the eyes (possibly due to mRNA instability) was confirmed by RT-PCR (Fig. 2d).

### The OMR test using monochromatic lights from the OLS, the O-O test

**Fig. 3:**
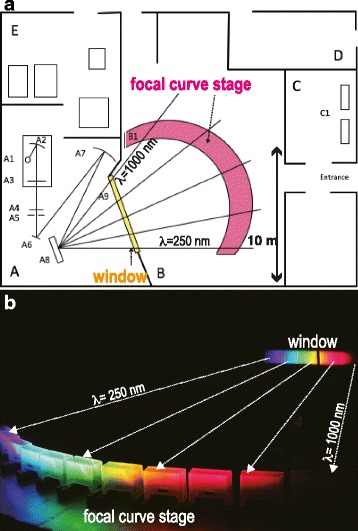
The Okazaki large spectrograph (OLS). (**a**) Layout of the OLS room (modified from [32]). A, monochromator room; B, irradiation room; C, sample preparation room; D, staff room; E, power supply room. A1, 30 kW xenon lamp; A2, rotatable condensing mirror; A3, shutter; A4, the heat-absorbing filter; A5, entrance slit; A6, plane mirror; A7, condensing mirror (110 × 110 cm, curvature radius of 9 m); A8, double-blazed plane grating (90 × 90 cm); A9, window; B1, focal curve stage; C1, incubators. Basically, the monochromatic lights are radially and horizontally irradiated from A8 to B1 where the OMR tests were performed. (**b**) The actual monochromatic lights irradiated in the OLS room. Plastic boxes were placed on the focal curve stage (bottom). The window (top right) is fully opened and all other lights in the OLS room were turned off. The light from the xenon lamp was thus dispersed into a contiguous spectrum from λ = 250 nm to λ = 1,000 nm. The intensities of the monochromatic lights at representative wavelengths are shown in Table 1

**Fig. 4:**
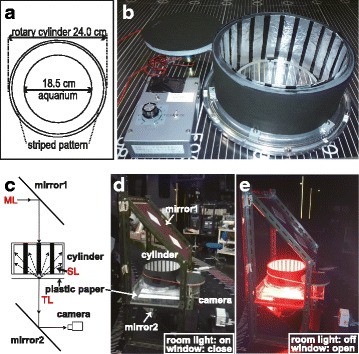
The equipment used for the O-O test. (**a**) and (**b**) Layout and appearance of the rotary-cylinder device. A glass aquarium was placed at the center of a rotary cylinder with vertical black-white stripes on the inner wall (in this picture, the stripes consist of aluminum-foil strips on Indian black ink for experiments under ultraviolet light; see text). The cylinder can be rotated by an electric motor at a constant speed (6 rpm). (**c**) Layout of the experimental platform for irradiation and behavior recording during the O-O test. The horizontal monochromatic light (ML) is reflected on the first mirror (mirror1) to illuminate the rotary-cylinder device from the top. The light goes through the aquarium, a white plastic paper under the aquarium, and the transparent acrylic base of the rotary-cylinder device (not shown), and this transmitted light (TL) is then horizontally reflected by the second mirror (mirror2), which is recorded by a digital CCD camera (ORCA-R2; Hamamatsu Photonics). The monochromatic lights are parallel lights and show the shadows of the fish in the aquarium clearly on the plastic paper (see Fig. 5), which we observed for the behavioral analyses. A part of the monochromatic light is scattered by the plastic paper, and these scattering lights (SL) can irradiate the inside of the cylinder. (**d**) and (**e**) Actual appearance of the experimental platform under the ceiling lights in the OLS room (**d**) and monochromatic red light (**e**). A flashlight was also used when taking these pictures

The *lws* ^*−*^ mutants should have difficulties in perceiving light at long wavelengths (red lights) due to lack of functional LWS opsins in the retina. To confirm this, we compared their behavioral responses (OMR) under red light. As a source of red monochromatic light that could illuminate the entire aquarium and surrounding stripes with sufficient intensity, the OLS in the National Institute for Basic Biology in Japan was ideal (Fig. 3). This spectrograph horizontally disperses a series of ultraviolet, visible, and infrared lights with wavelengths from 250–1,000 nm to a U-shaped focal surface of about 10-m wide by diffracting white light (30 kW) from an electrode water-cooled xenon short arc lamp (Fig. 3a). In short, this machine is capable of creating a rainbow in a dark room (Fig. 3b). Monochromatic lights are highly purified and have a half bandwidth (4 ~ 5 nm) that is less than that of ordinary LED lights (>20 nm). Taking the diameter of the aquarium (18.5 cm) into consideration (Fig. 4a), however, it should be noted that the aquarium and cylinder receive monochromatic light of the intended wavelength only in the center; i.e., monochromatic lights of ±10 nm wavelengths are irradiated at either side of the cylinder.

To carry out the OMR test in OLS, termed the O-O test, we made an automatic rotary cylinder with black/white stripes (Fig. 4b). In addition, we made a mobile light-reflecting device capable of irradiating the monochromatic light perpendicularly into the aquarium (Fig. 4c–e). Namely, a mirror on top (mirror1 in Fig. 4c) reflects the horizontal monochromatic light flux into a vertical direction to irradiate the aquarium containing the fish and a plastic white paper beneath it. The paper partially reflects (scatters) the light and irradiates the black/white stripes inside the cylinder. The light which passes through the plastic paper is then reflected horizontally to the camera by another mirror (mirror2 in Fig. 4c) for video-recording.

**Fig. 5:**
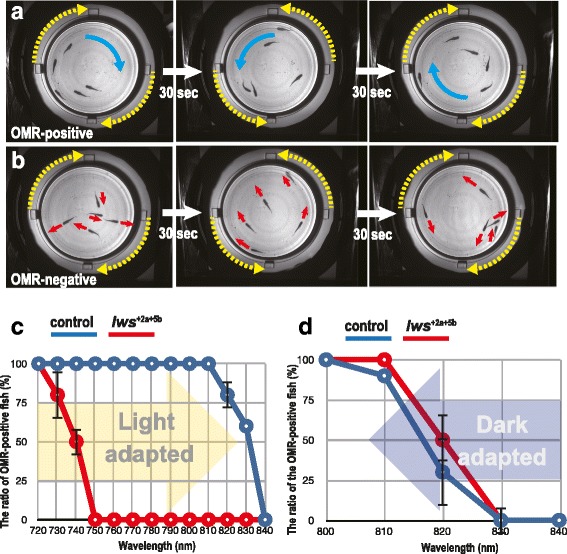
The optomotor response (OMR) under monochromatic lights after light or dark adaptation. (**a**) and (**b**) Validation criteria during the O-O test: a, OMR-positive; b, OMR-negative. Yellow dotted arrows indicate directions of the cylinder rotation. Blue arrows indicate all fish in the aquarium (*n* = 5) are following the stripes, whereas red arrows indicate the fish are swimming independent of the cylinder rotation. We switched the direction of cylinder rotation every 30 s and observed if all the fish started following within 10 s. These pictures are frames of movies recorded at λ = 760 nm using the control (**a**) and *lws* ^*−*^ (**b**) fish. (**c**) Summary of the results using light-adapted fish. We started the OMR test at λ = 720 nm, where both the control (blue) and *lws* ^*−*^ (red) fish had followed the stripes in the preceding experiments (data not shown). After each test, we repeated it under monochromatic light with a wavelength 10 nm longer until λ = 840 nm (yellow arrow), where all the fish had stopped following the stripes. Each circle is an average of two independent experiments. Error bars are attached when applicable. Note that the *lws* ^*−*^ mutants stopped following stripes by λ = 750 nm, whereas the control fish continued following until λ = 830 nm. (**d**) Summary of the results using dark-adapted fish. We started the OMR test at λ = 840 nm, where none of the control (blue) or *lws* ^*−*^ mutant (red) fish had followed the stripes in the preceding experiments (data not shown). After the test, we repeated it under monochromatic light with a wavelength 10 nm shorter until λ = 800 nm (blue arrow), where all the fish started following the stripes. Note that both the control and *lws* ^*−*^ mutant fish started to respond to lights at λ = 820 ~ 830 nm. That is, the *lws* ^*−*^ mutants could behaviorally respond to monochromatic lights at λ = 750 ~ 830 nm only when they had been dark-adapted



Using this system, we tested OMR of the control and *lws* ^*−*^ fish at every 10 nm of wavelengths from 700 nm to 900 nm. During the test, the cylinder was rotated at 6 rpm and the rotating direction was switched every 30 s. If the fish seemed as if they were following the stripes (see Additional files 1–3: Movies S1–S3), we judged that the OMR (or OMR and schooling; [25]) was positive and that the fish in the aquarium could see (perceive) the monochromatic light (Fig. 5a). Contrary, if the fish did not show following behavior (see Additional files 4–6: Movies S4–S6), we judged that OMR was negative and that the irradiated monochromatic light was invisible to the fish (Fig. 5b). We found that the control medaka could follow the stripes up to λ = 820 ~ 830 nm, but not at λ = 840 nm (Fig. 5c, blue line). In contrast, the *lws* ^*−*^ mutants (*lws* ^+2a+5b^) followed the stripes up to λ = 720 ~ 740 nm, but not at λ = 750 nm (Fig. 5c, red line), demonstrating that the double-frameshift mutations on the *LWSa* and *LWSb* genes successfully inhibit the perception of red light.

**Additional file 1: Movie S1.** The OMR of control medaka at λ = 720 nm. Five control fish immediately followed the rotating stripes, whenever the direction of rotation was changed. (MOV 5599 kb)




**Additional file 2: Movie S2.** The OMR of *lws* ^*−*^ mutants at λ = 720 nm. Five *lws* ^*−*^ mutants followed the stripes similarly to the control fish. (MOV 3587 kb)




**Additional file 3: Movie S3.** The OMR of control medaka at λ = 760 nm. Five control fish followed the rotating stripes as they did at λ = 720 nm. (MOV 5123 kb)




**Additional file 4: Movie S4.** The OMR of *lws* ^*−*^ mutants at λ = 760 nm. Five *lws* ^*−*^ mutants did not follow the rotating stripes. Although they seemed to respond to the (sound of) cylinder rotation at the beginning, their swimming did not seem to be affected by the direction of the cylinder rotation. (MOV 10174 kb)




**Additional file 5: Movie S5.** The OMR of control medaka at λ = 850 nm. Five control fish did not follow the rotating stripes. Similar to the *lws* ^*−*^ mutants at λ = 760 nm, their swimming seemed to be independent of the cylinder rotation. Note that many ripples were made by the fish on the water surface, which was rarely observed when fish were following the rotating stripes (see S1–S3 Movies). (MOV 9346 kb)




**Additional file 6: Movie S6.** The OMR of *lws* ^*−*^ mutants at λ = 850 nm. Five *lws* ^*−*^ mutants did not follow the rotating stripes. (MOV 3509 kb)


### F_2_ screening by the O-O test at λ = 760 nm

To evaluate the accuracy of our manual judgments (OMR-positive or OMR-negative) in the O-O test, we conducted screening of F_2_ fish (obtained by intercrossing heterozygous *lws* ^*−*^ mutants) with unknown genotype by the O-O test. We individually observed OMR of each F_2_ fish at λ = 760 nm that could be perceived by the control, but not the *lws* ^*−*^ fish (Fig. 5c). In the *lws* ^*+2a+5b*^ line (*n* = 29), we judged that 23 of the F_2_ fish were OMR-positive and six were OMR-negative. After this screening, we genotyped each fish and found that 97% (28/29) of our judgments were correct; i.e., all the OMR-positive fish were homozygotes of the wild-type alleles or heterozygotes of the wild-type and *lws* ^*+2a+5b*^ alleles (nine and fourteen F_2_ fish, respectively). Five of the six OMR-negative F_2_ fish were homozygous for the *lws* ^*+2a+5b*^ allele, but one was a heterozygote. We suspect that this was because we had overlooked the indifference of this F_2_ to the rotating stripes. Fish did not always show OMR whenever the stripes were rotating. Sometimes, they seemed to lack an interest in the rotating stripes and were pecking at something at the bottom of the aquarium or intently swam along the wall of the aquarium. In these cases, we put such restless fish back in a stock tank and repeated the O-O test, but may have overlooked the fish for which we made the misjudgment.

Similar results were obtained in the tests using F_2_ fish of the *lws* ^−1a-19b^ and *lws* ^−2a-19b^ strains. A total of 48 F_2_ fish were individually analyzed by the O-O test, and we judged that 25 and 23 were OMR-positive and OMR-negative, respectively. Again, all the OMR-positive fish had at least one wild-type allele, whereas 14 of the OMR-negative fish did not. Thus, we made nine misjudgments (the accuracy of judgments was 81.3% = 39/48) most likely by overlooking the indifference of fish to the rotating stripes. Given that none of the *lws* ^*−*1a-19b^ and *lws* ^*−*2a-19b^ F_2_ fish showed OMR (note that the OMR-positive fish never included the *lws* ^*−*^ mutants), these mutants could also have similar defects in red-light sensitivity as the *lws* ^*+2a+5b*^ mutants.

### The O-O tests using dark-adapted fish

Finally, we tested whether the rod vision of the *lws* ^*−*^ mutants is affected by using dark-adapted fish. We kept the control and *lws* ^*−*^ (*lws* ^*+2a+5b*^) fish in black boxes for more than 2 h to induce dark adaptation, and started the test at λ = 900 nm. The OMR could not be detected until λ = 840 nm in either the control or mutant strains, but both started to respond at λ = 830 ~ 820 nm (Fig. 5d). These results clearly demonstrate that spectral sensitivity (visible light) is different between light- and dark-adapted conditions in the *lws* ^*−*^ mutants, whereas control fish can perceive red light in both conditions. Therefore, the double-frameshift mutations on the *LWSa* and *LWSb* genes could specifically suppress the LWS-dependent cone vision, and the RH1-dependent rod vision seemed to remain intact.

We also tested OMR of the wild-type medaka (the HNI strain) and obtained results similar to those of the control fish in both the light- and dark-adapted conditions (data not shown).

## Discussion

### Producing color-blind fish lines: Simultaneous loss of evolutionally multiplied genes using the CRISPR/Cas9 system

In this study, we made red color-blind medaka by disrupting all paralogous *LWS* genes using a single sgRNA, which was designed in common sequences of *LWSa* and *LWSb*. To reduce off-target mutation, the length of all the sgRNA targeting sequences was designed to be 18 nt [26]. We showed that the appropriate sgRNA induced double-ins/del mutations on both paralogous genes in more than 60% of G_0_ adults (Fig. 1c), and nearly half of F_1_ inherited double-frameshift mutations (Fig. 2c). Thus, this method (particularly that using sgRNA-4) was quite efficient at inducing the simultaneous loss of genes with consensus sequences.

### A simple but sophisticated spectral sensitivity test for small fish: the OMR test using the OLS (the O-O test)

To measure the behavioral spectral sensitivity of small fish, we developed an OMR test using the OLS. Comparing the primary OMR experiments using the OLS [12], our system should have some advantages. First, the light conditions are more natural. We irradiated the aquarium from the top (Fig. 4c–e), whereas Hasegawa showed fish monochromatic light horizontally through striped slits on a rotating drum. Second, fish responded to the rotating stripes much sooner (<10 s as natural speed) than they did in the previous work (>2 min), even under the near-infrared light (λ = 830 nm). This could partially be because the photon flux density (brightness) was much higher in this study (about 50 μmol/m^2^s; Table 1) than that in the previous work using band-pass and neutral-density filters (about 0.3 μmol/m^2^s). Third, a new mobile device for vertical irradiation and video-recording (Fig. 4c–e) enabled convenient operations in fine steps (every 10 nm) in a wide-range wavelength. Fourth, we could obtain clear images of moving fish by recording their shadows at the bottom of the aquarium (see Methods). This enabled the real-time and remote (indirect) observation of OMR occurring in the aquarium, which will become necessary in the O-O test under ultraviolet lights for analyzing the spectral sensitivity of blue or violet color-blind fish.

It is worth mentioning here that the O-O test in the ultraviolet region has some technical problems to overcome. First, ultraviolet light provokes fluorescence of the rotating devices. Second, the glass mirrors and glass aquarium absorb (i.e., do not efficiently transmit or reflect) ultraviolet light (λ < 320 nm). Third, experiments under strong ultraviolet lights may cause harmful effects in the retina (spectral sensitivity) of fish. We are currently trying to solve these issues by selecting non-fluorescent papers or inks, replacing the glass devices by crystal ones, etc.

### Usefulness of this system: fish models for studying visual systems and vision-dependent behavior

**Fig. 6:**
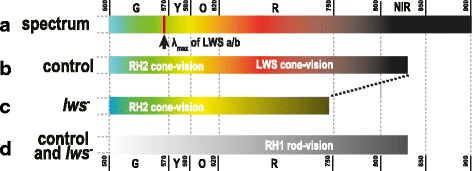
The behavioral spectral sensitivities of the control and *lws* ^*−*^ medaka. (**a**) The schematic diagram of wavelengths and colors of light at λ = 500 ~ 900 nm. The lights gradually change color from green (G), yellow (Y), orange (O), to red (R), and then become invisible (near-infrared light, NIR) to humans. The color medaka actually provokes in the brain from these monochromatic lights is unknown. (**b** and **c**) Visible lights for the light-adapted control (**b**) and *lws* ^*−*^ (**c**) medaka. The fish with wild-type LWS opsins could follow the stripes up to λ = 820 ~ 830 nm, and the *lws* ^*−*^ medaka could follow them up to λ = 730 ~ 740 nm. This ~90-nm decrease of the longest wavelength of visible light in the photopic vision in the *lws* ^*−*^ medaka demonstrates that; (1) the LWS-dependent cone vision, which covers up to λ = 830 nm, was severely suppressed in the *lws* ^*−*^ mutants, and (2) the RH2-dependent cone vision should cover up to λ = 740 nm. (**d**) Visible lights for the dark-adapted control and *lws* ^*−*^ medaka. Both medaka can perceive monochromatic light up to λ = 830 nm, suggesting that *lws* ^*−*^ medaka retain the ordinary RH1-dependent rod vision

In the experiments by Hasegawa [12], the wild-type medaka showed OMR at λ = 400, 500, and 600 nm in both the light- and dark-adapted conditions. Adult zebrafish are also known to respond behaviorally to light at λ = 340 ~ 640 nm [27]. Light with much longer wavelengths (λ = 700 ~ 900 nm) was used in this study, which revealed that the control (and wild-type) medaka could perceive and respond behaviorally to monochromatic light with wavelengths of up to 820 ~ 830 nm (Fig. 6). This level of red or near-infrared light has not been used in previous behavioral, optokinetic, or physiological studies; typically, light of λ < 700 nm has been used. We expect that fish could have perceived light at even longer wavelengths if the experimental conditions had been further optimized (e.g., pattern and speed of the rotating stripes, distance between fish and the stripes, smoothness of the aquarium wall, brightness of the light, etc.). Considering that the λ_max_ of medaka LWSa and LWSb is about 560 nm [7], the LWS vision in medaka seemed to cover an unexpectedly wide range of wavelengths (λ = 290 ~ 560 ~ 830 nm) (Fig. 6b). That is, light-adapted medaka may perceive and behaviorally respond to ultraviolet lights by using LWS cones alone.

Similarly, the results of the O-O test using the *lws* ^*−*^ mutants indicated that the green opsins of medaka (RH2a, RH2b, and RH2c) should also cover a rather wide range of wavelengths. The shortest and longest λ_max_ of RH2s is 452 and 516 nm, respectively [7], but the light-adapted *lws* ^*−*^ fish showed OMR of up to λ = 740 nm, most likely via the RH2 vision. Thus, the RH2 vision may cover λ = 230 ~ 450 ~ 520 ~ 740 nm (Fig. 6c). We are currently establishing *rh2* ^*−*^ medaka strains, but expecting that their spectral sensitivity might not differ from that of control fish, because the range of wavelengths which RH2a, RH2b, and RH2c cover largely overlap with those covered by LWSa and LWSb (and probably also SWS2a and SWS2b). Different methods or the establishment of the *lws* ^*−*^ *; rh2* ^*−*^ double mutants (actually, the *lwsa* ^*−*^ *; lwsb* ^*−*^ *; rh2a* ^*−*^ *; rh2b* ^*−*^ *; rh2c* ^*−*^ quintuple mutant) might be necessary to demonstrate that RH2 vision is indeed suppressed by the *rh2* ^*−*^ mutations.

Another important finding in this study is that rod (RH1) vision surely exists in medaka. The fact that the light-adapted *lws* ^*−*^ mutants did not show OMR at λ > 740 nm (Fig. 5c) indicates that their rods, in addition to their dysfunctional LWS cones, contribute little to photopic vision. In addition, the fact that the dark-adapted *lws* ^*−*^ mutants did show OMR at λ > 740 nm, as in the wild type (Fig. 5d), indicates that the mutants had retained fully functional rods and the LWS cones have only negligible effects in scotopic vision.

Previous ERG-based studies, however, indicated that the LWS cones contribute to scotopic vision in goldfish [28] and giant danio [29], whereas the contribution is negligible in a few marine fish (i.e., snapper, grunt, and grouper [30]). These (and other) ERG studies were conducted at wavelengths of λ = 700 nm or less, whereas we analyzed scotopic vision at λ > 740 nm. Because of these differences in species, experimental methods (physiological vs. behavioral), and wavelengths (shorter vs. longer than 700 nm), it is difficult to understand what these consistent and inconsistent results actually mean. Nevertheless, our present results clearly demonstrated that the rod vision of medaka is much more sensitive than the cone vision at λ = 730 ~ 830 nm and that scotopic vision could almost solely rely on rods, at least in these wavelengths.

### Future use of the O-O test and color-blind medaka: analysis of fish models for studying color perception in vertebrates

There has not been fish model for representing color blindness of humans, except for the zebrafish mutant that does not optokinetically and physiologically respond to red light, but this mutant is a recessive lethal and the mutation is on neither of the *rod-* or *cone-opsin* genes [27]. The *lws* ^*−*^ medaka established in this study should be the first model for so-called color blindness in fish.

We expect that the *lws* ^*−*^ mutants would be useful for developing gene-therapeutic methods for protanopia [31]. This is because the red-light sensitivity of *lws* ^*−*^ fish is markedly different from that of wild-type fish (Fig. 6) and because the recovery of the red-light sensitivity by gene delivery would be revealed easily, non-invasively, and reliably by the O-O test (Fig. 5). This simple system reported here could additionally establish green, blue, and violet color-blind medaka lines. The ultimate color-blind line, which lacks all cone opsins, could also be produced by crossing single color-blind medaka. Using the variety of such lines, the function of cone opsins and systems for color perception may be investigated further.

## Conclusion

In this work, we established a simple but sophisticated system for making color-blind fish and testing their spectral sensitivity. The CRISPR/Cas9 system enabled us to induce simultaneous mutations in paralogous genes by designing sgRNA at consensus sequences. The OMR test using the OLS, termed the O-O test, enabled us to evaluate behavioral spectral sensitivity under light- and dark-adapted conditions. Further establishments and analyses of other cone-opsin mutants using this system would reveal the mechanisms for color perception in this species as well as other non-mammalian vertebrates, and would likely be useful for the advancement of genetic or regenerative methods to treat human color blindness.

## Abbreviations

λ_max_: Peak absorption spectrum
CRISPR/Cas9: Clustered regularly interspaced short palindromic Repeat/CRISPR-associated protein 9
LWS: Long-wavelength-sensitive
OLS: Okazaki large spectrograph
OMR: Optomotor response
O-O test: OMR under OLS test
RH: Rhodopsin
SWS: Short-wavelength-sensitive.
